# Concise and Straightforward Asymmetric Synthesis of a Cyclic Natural Hydroxy-Amino Acid

**DOI:** 10.3390/molecules191219516

**Published:** 2014-11-26

**Authors:** Mario J. Simirgiotis, Javier Vallejos, Carlos Areche, Beatriz Sepúlveda

**Affiliations:** 1 Laboratorio de Productos Naturales, Facultad de Ciencias Básicas, Universidad de Antofagasta, Avenida Universidad de Antofagasta 02800, Casilla 170, Antofagasta 1240000, Chile; 2 Departamento de Química, Universidad Católica del Norte, Av. Angamos 610, Antofagasta 1240000, Chile; E-Mail: jvallejos01@ucn.cl; 3 Departamento de Química, Facultad de Ciencias, Universidad de Chile, Casilla 653, Santiago 7800024, Chile; E-Mail: areche@uchile.cl; 4 Departamento de Ciencias Químicas, Universidad Andrés Bello, Campus Viña del Mar, Quillota 980, Viña del Mar 2520000, Chile; E-Mail: bsepulveda@uc.cl

**Keywords:** pipecolic acid, total synthesis, piperidine, amino acids

## Abstract

An enantioselective total synthesis of the natural amino acid (2*S*,4*R*,5*R*)-4,5-di-hydroxy-pipecolic acid starting from D-glucoheptono-1, 4-lactone is presented. The best sequence employed as a key step the intramolecular nucleophilic displacement by an amino function of a 6-*O*-*p-*toluene-sulphonyl derivative of a methyl d-arabino-hexonate and involved only 12 steps with an overall yield of 19%. The structures of the compounds synthesized were elucidated on the basis of comprehensive spectroscopic (NMR and MS) and computational analysis.

## 1. Introduction

Cyclic hydroxy-amino acids are adequate building blocks for the development of new therapeutic drug candidates [1,2,3]. These compounds are β-turn inducers in the synthesis of peptides [4] and good enzyme inhibitors by themselves [5] as are several of their derivatives, such as deoxy-galactono-jirimicin derivatives [6,7,8]. Indeed, stereoselective synthesis of hydroxy-amino acids is a field that has been growing during the last decades because of the potential biological activity of some representatives of this class of compounds, mainly as enzyme inhibitors [5,9,10,11,12]. Particularly, conformationally constrained hydroxy-amino acids such as the hydroxylated pipecolic acids are very interesting target molecules [12,13]. For instance, the compound (2*S*,4*R*)-4-hydroxy-pipecolic acid is a natural product that has been isolated from *Calliandra pittieri* and *Stophantus scandeus*[14], and the *cis*-5-hydroxy-substituted pipecolic acid skeleton is frequently found in alkaloids from microorganisms and also in plants (febrifugine, pseudoconhidrine) [15,16]. Furthermore, a tri-hydroxy-pipecolic acid isolated from the seeds of *Baphia racemosa* proved to have specific human liver beta-glucosidase inhibitory activity [11]. A couple of years ago a stereoselective synthesis of *N*-Boc-protected *cis*-(2*R*,3*S*)-3-hydroxy-pipecolic acid, starting from d-glucose, was described [17]. In the literature some synthesis of 5-hydroxy-pipecolic acid derivatives and their analogues is also described [18,19,20]. A record of an enantioselective synthesis of (2*R*,4*R*)- and (2*S*,4*R*)-4-hydroxy-pipecolic acids from commercial ethyl (*R*)-4-cyano-3-hydroxy-butanoate was published [21], while the synthesis of the latter compound was also performed starting from d-glucoheptono-1,4-lactone [22]. Moreover, recently two efficient and complementary routes have been described for the synthesis of 4,5 di-hydroxy-pipecolic acids from cis (4*S*,5*R*)- and (4*R*,5*S*)-4,5-dihydroxy-γ-valerolactams [23]. In this work we describe another short, convenient and enantioselective synthesis of (2*S*,4*R*,5*R*) 4,5 di-hydroxy-pipecolic acid starting from **2** as chiral template. This target cyclic amino acid is a natural product that has been isolated from the leaves of *Julbernardia paniculata* [24] and synthesized from the precursor L-baikiaine, isolated from the aerial parts of *Derris eliptica* [25].

Mario Simirgiotis has worked previously on the synthesis of small molecules starting from sugars as chiral templates [26] and embarked on the synthesis of this compound several years ago (under the mentorship of Dr. Oscar Varela, at the University of Buenos Aires, Argentina), but the results were unpublished.

## 2. Results and Discussion

### 2.1. Retrosynthetic Analysis for the Synthesis of Compound **1**

**Scheme 1:**
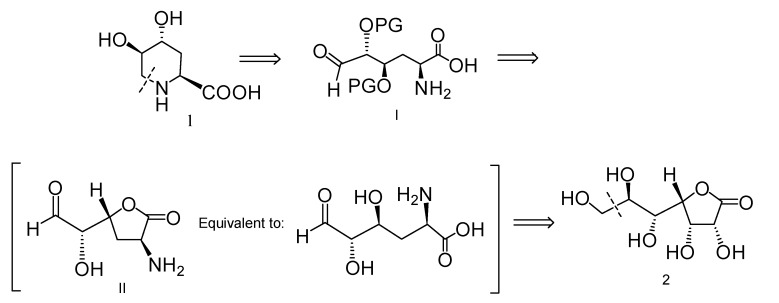
Retrosynthetic analysis of the target amino acid **1**.

We reasoned (Scheme 1) that **1** can be synthesized by imine hydrogenation after direct carbonyl addition by the amino group at C-2 (synthon **I**), a strategy that was previously employed for the synthesis of (2*S*,4*R*) 4-hydroxypipecolic acid [22]. Synthon **I** can readily be obtained through *O*-protection of α amino lactone **II**. Key intermediate **II** can be in turn obtained from commercially available **2** as a chiral template via a series of elimination of the HO-3 subsequent oxidation, and conversion of the HO-2 into NH_2_.

### 2.2. Total Synthesis of Compound **1**

**Scheme 2:**
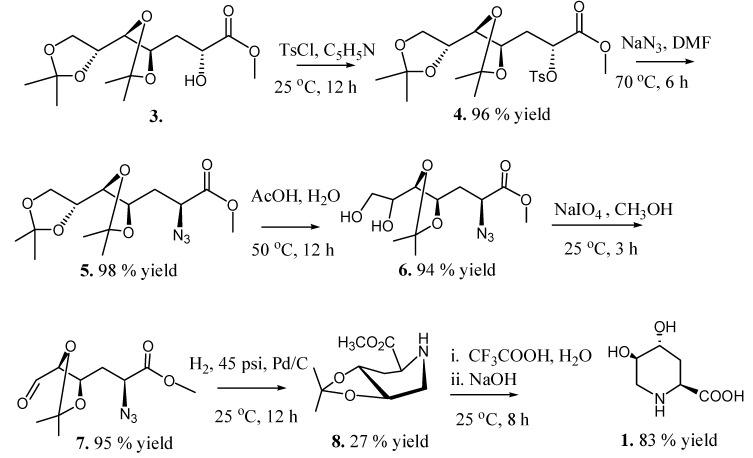
Synthesis of compound **1** from **3**.

We started with commercially available and inexpensive d-glycero-d-gulo-heptono-1,4-lactone (**2**) using a previously published methodology [27], which, on peracetylation and β-elimination of the acetate group on C-3, followed by hydrogenation, *O*-deacetylation, and isopropylidenation of the resulting aldonolactone with 2,2-dimethoxypropane, yielded 3-deoxy methyl ester **3**, which has the desired (4*R*,5*R*) configurations (Scheme 2). The free hydroxyl group at C-2 of **3** was sulfonylated with tosyl chloride in pyridine to give the tosylate derivative **4** in 12 h with 96% yield. Spectroscopic data for compound **4** resembled the distorted planar zigzag conformational preference in solution as previously reported for compound **3** [27] with an *anti* disposition for H-5 and H-6 (*J*_5,6_ 8.3 Hz). NOE correlations between H-2–H-3, H-3–H-4, H-3–H-5, and H-4–H-6 in the NOESY spectrum of **4** also corroborated that conformation.

**Figure 1:**
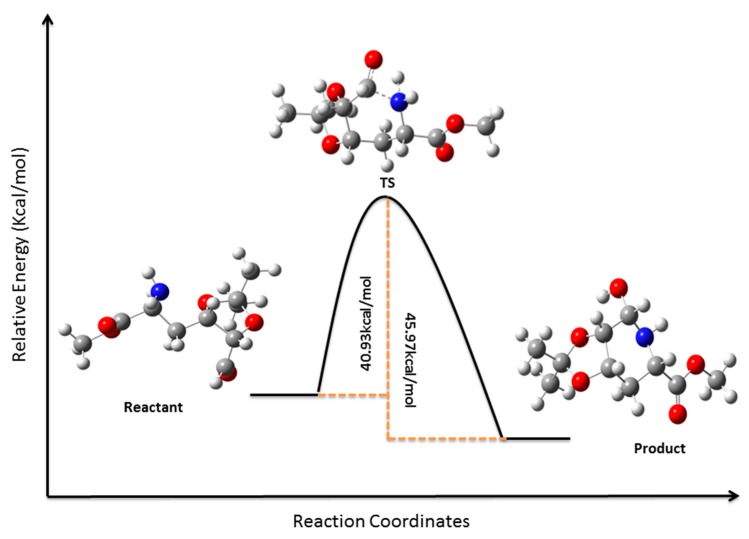
Minimum energy conformations, activation energy, and transition state for the intramolecular nucleophilic attack of amine on the aldehyde of intermediate **7a** (reactant) to obtain **8** (product). (DFT, Gaussian 9.0) [28].

**Scheme 3:**
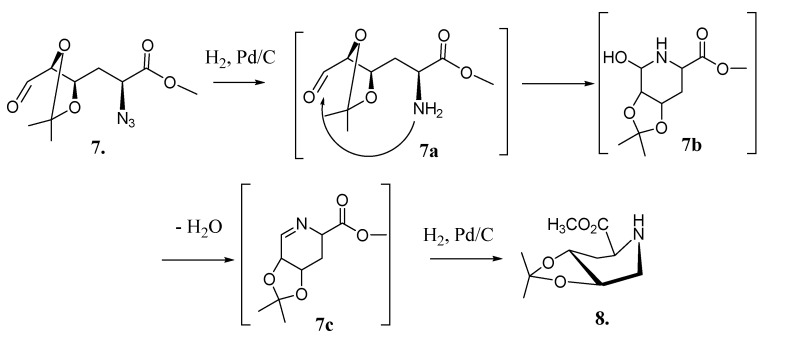
One pot synthesis of piperidine **8** from azide **7**.

Treatment of tosylate **4** with sodium azide in DMF produced the desired inversion of C-2 configuration affording azide **5** in 87% yield. Selective hydrolysis of the isopropylidene terminal group of **5** yielded diol **6**, which on degradative oxidation of the 1, 2 diol system using sodium periodate produced aldehyde **7**. Aminolysis of **7** in a “one pot” procedure (Scheme 3, Figure 1) yielded key compound **8**through a sequence of catalytic hydrogenation of the azide group, nucleophilic attack of the resulting amine on the aldehyde (**7a**), dehydration of the resulting alcohol **7b** to the imine **7c**, and reduction of the imine **7c** to give the expected piperidine **8**, which crystallized from hexane-EtOAc 10:1 (mp 119 °C, [α]_D_ −47.62). In order to demonstrate this reaction and try to understand the low yield (27%) obtained, an Internal Reaction Coordinate (IRC) calculation was performed. An IRC calculation allows one to map out a reaction pathway by integrating the intrinsic reaction coordinate. This method examines the reaction pathway proposed, leading to a transition state in a map of the potential energy surface. The results of this calculation are showed in Figure 1. The transition state (TS) was found with an imaginary frequency, demonstrating that this is, as expected, a first-order and six-membered cyclic TS. 

**Figure 2:**
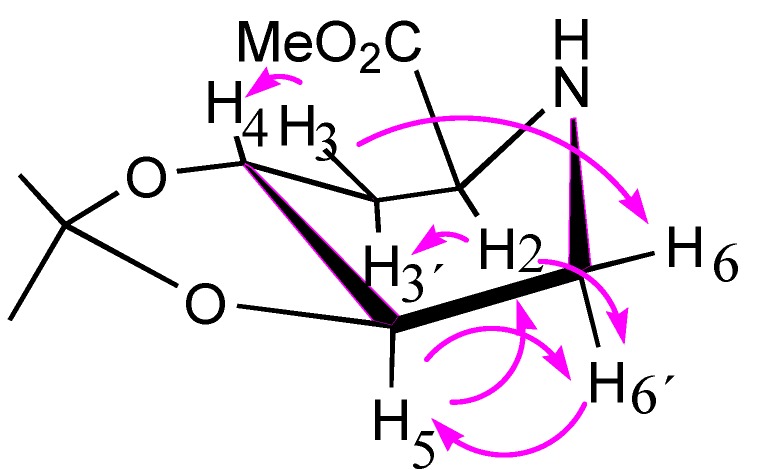
Key NOE correlations for compound **8**.

Coupling constant analysis of the ^1^H-NMR data of compound **8** suggested *anti* dispositions between H-4 and H-5 (*J*_4,5_ ≈ 6.0), H-5 and H-6 (*J*_5,6_ ≈ 6.0, *J*_5,6'_ = 2.8 Hz), and H-2 and H-3 (*J*_2,3'_ = 4.4, *J*_2,3_ = 8.8 Hz), suggesting a boat (^4^B_N_) conformation, which was confirmed by a NOESY experiment (NOE cross peaks between H-3 and H-4, H-3' and H-2, and H-5 with H-3' and H-6' (Figure 2)).

**Figure 3:**
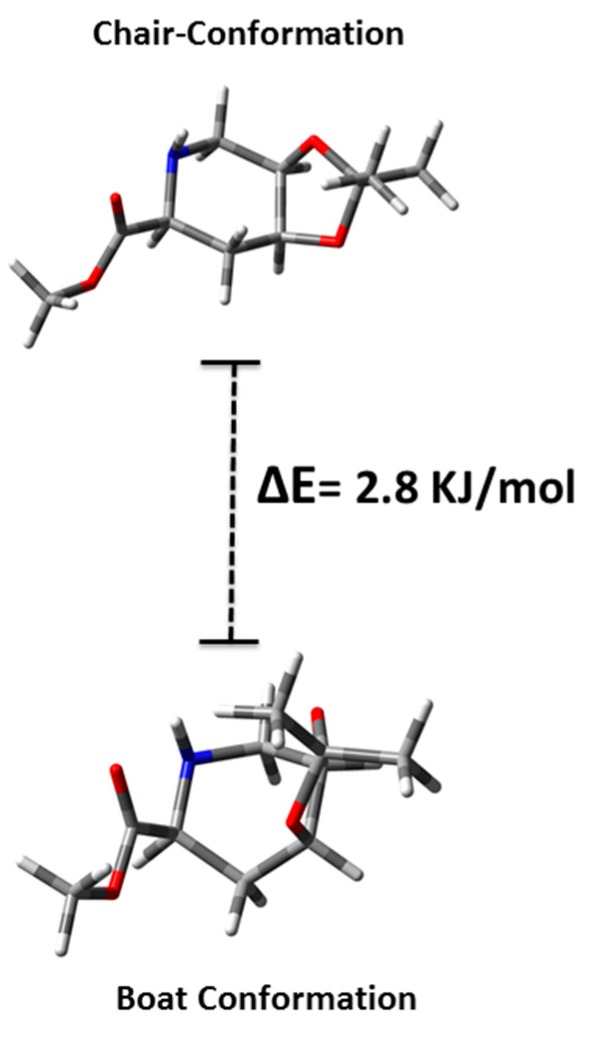
Minimum energy conformations of target compound **8** calculated by DFT (Gaussian 9.0) [28].

Furthermore, an intense cross-peak observed between H-6' and H-2 confirmed the *S* configuration for C-2. The correlations were corroborated by HMQC and HMBC spectra. Density Functional Theory (DFT) calculations (Gaussian 9.0) [28] indicated that compound **8** has two minimum-energy conformations (boat and chair) but the boat conformation (^4^B_N_) is 2.8 KJ/mol more stable (Figure 3), which is in concordance with the spectroscopic data found for this compound. The isopropylidene group of amino-acid **8** was removed by stirring with trifluoracetic acid-water (1:1) for 8 h at room temperature, while removal of the remaining ester function at C-1 was achieved by subsequent alkaline treatment with NaOH 1 M to yield the target cyclic amino acid **1**.

The preparation of key compound **8** from **7** (Scheme 3) resulted in a disappointing 27% yield. We assayed different concentrations of catalyst, solvent, hydrogen pressure, and reaction times (Table 1), but in all cases after consumption of all starting material (monitored by TLC) we obtained compound **8** (yields below 27%) and a mixture of polar compounds, according to ^1^NMR a mixture of adducts produced by intermolecular addition of the amine group at C-2 of one molecule of **7** to the aldehyde function at C-6 of another molecule of **7**. We assumed that the low yield obtained for this reaction could be attributed in part to the partial conformational impediment (Figure 1) produced by the isopropylidene group for the nucleophilic addition of the C-2 amine group to the aldehyde.

**Table 1:** Preparation of compound **8** by catalytic hydrogenation of aldehyde **7**.

Catalyst (Pd-C)%	Hydrogen Pressure (psi)	Time	Solvent	Yield (%) ^b^
10	20	8 h	EtOAc	23
10	45	8 h	EtOAc	25
10	45	24 h	EtOAc	26
15	45	48 h	EtOAc	27
15	30	48 h	EtOAc	27
20	30	24 h	EtOAc	27
25	45	24 h	EtOAc	27
10	30	48 h	EtOAc/MeOH	25
15	45	24 h	EtOAc/MeOH	24
15	30	8 h	EtOAc	23
20	30	24 h	EtOAc/MeOH	26
20	45	24 h	EtOAc/MeOH	25
20	45	24 h	MeOH	25

^b^ Isolated yields. All reactions were performed at 20 °C.

### 2.3. Synthesis of Compound **1** Starting from Azide **5**

**Scheme 4:**
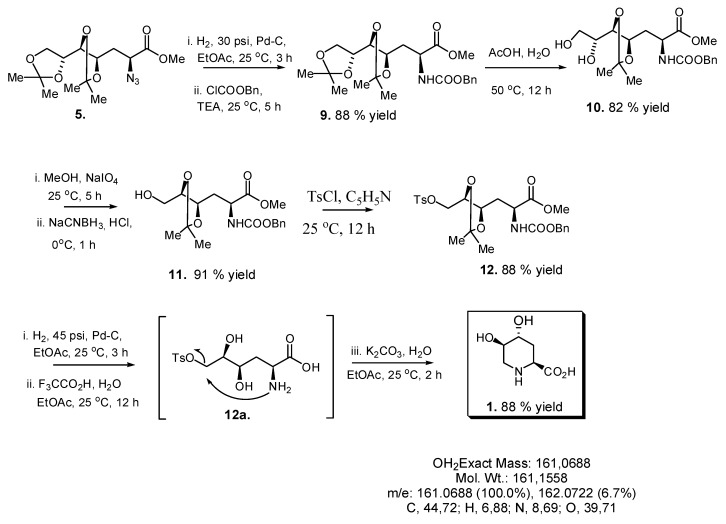
Synthesis of compound **1** from azide **5**.

The low yield obtained for key compound **8** from **7** prompted us to use the alternative synthetic sequence depicted in Scheme 4. Catalytic hydrogenation of **5** in EtOAc generated the amino group that was in turn protected with benzyl chloroformate to obtain carbamate **9** in 88% yield. Furthermore, the benzyloxycarbonyl was a convenient protective group for the amine group of this small class of sugar compounds, since a very stable carbamate derivative can be obtained and the protecting group can be easily removed by hydrogenolysis in high yields and short reaction times [22]. We considered that **10** was a convenient precursor of **1** that could be degraded to the aldehyde **11** and this compound could be properly reduced to the primary alcohol, which also has the right configuration and hydroxyl group for the functionalization with a convenient leaving group at C-6 to be displaced by the amino group at C-2, after removal of the benzyloxycarbonyl group. Thus, selective hydrolysis of the terminal isopropylidene group with a mixture of acetic acid-water at 50 °C over 12 h produced **10** in 82% yield, which could be converted to **11** using the procedure applied previously in the synthesis of **7**, followed by reductive amination with NaBH_3_CN at pH = 4 in 4 h with 91% yield. The tosylate **12** was then prepared as explained above to generate the necessary leaving group for the intramolecular nucleophilic displacement by the amino group.

**Figure 4:**
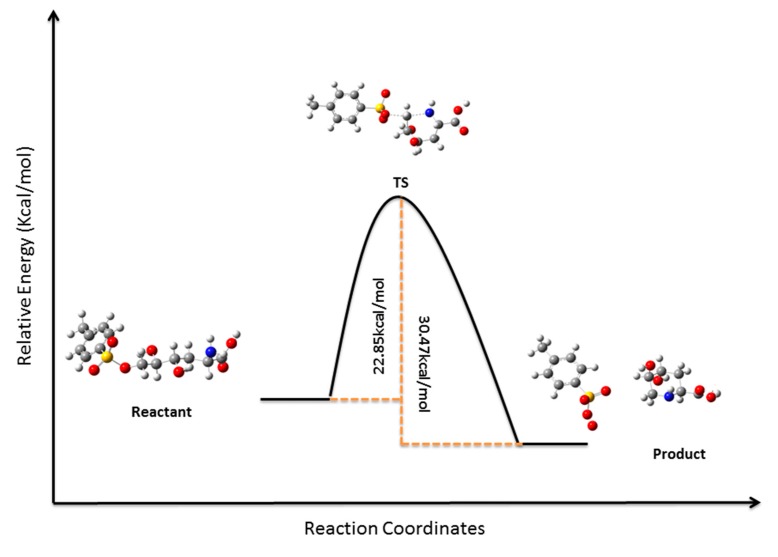
Minimum energy conformations, activation energy, and transition state for the intramolecular ring closure of intermediate **12a** to obtain **1** (DFT, Gaussian 9.0) [28].

Deprotection of the amine group of **12** was performed with 10% palladium on charcoal under hydrogen atmosphere (45 psi) in EtOAc for 12 h, and after filtration and evaporation of the solvent, the isopropylidene and ester groups of the latter compound were removed by hydrolysis with trifluoracetic acid: water (1:1). The resulting crude product, after evaporation of the solvent, was dissolved in water and treated with K_2_CO_3_ for 2 h to produce the nucleophilic amine group necessary for the ring closure (Scheme 4 and Figure 4). As shown in Figure 4, the activation energy for the closure of intermediate **12a** (reactant) into **1** (product) is almost half that for the intramolecular nucleophilic attack of amine on the aldehyde of intermediate **7a** to obtain **8** (Figure 1). This can explain in part the different yield obtained for the mentioned reactions.

**Figure 5:**
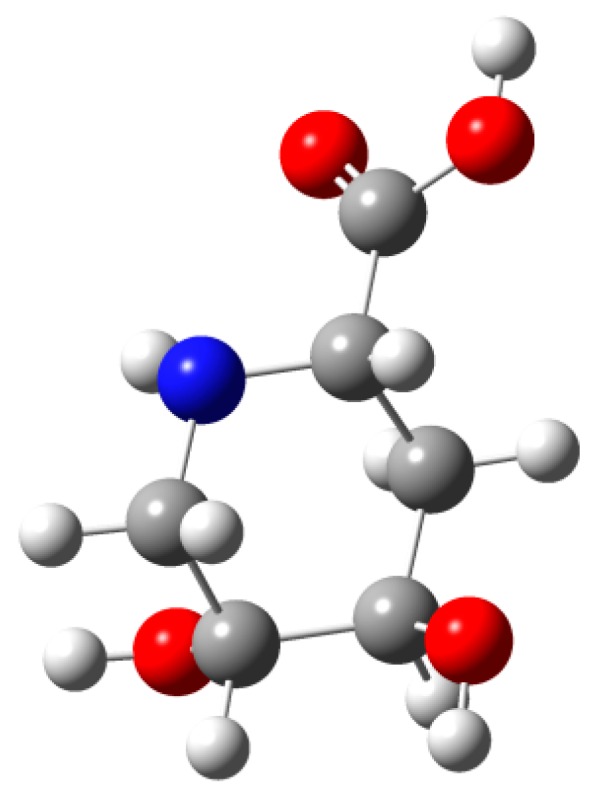
Minimum energy conformation of target compound **1** calculated by DFT (Gaussian 9.0) [28].

After 5 h of reaction only one product reactive to ninhydrin was obtained, and the solution was filtrated, acidified to pH = 6, and loaded onto an ion exchange column (Dowex 50W-H^+^ resin), which was rinsed with water and eluted with pyridine 0.1 N to obtain the target syrupy pipecolic amino-acid **1** with an [α]_D_ −5.4, and HRESIMS: [M+H]^+^ = 162.0763, without epimerization in 83% yield. ^13^ NMR spectra for **1** showed the expected six carbons, including one carboxylic acid (δ 173.35), three oxygenated methynes (C-2, C-4, and C-5 at δ 53.5, 65.4, and 64.9, respectively), and two methylene carbons (C-3 and C-6, at δ 28.4 and 44.2). All assignments were performed by careful analysis of the COSY, NOESY, HMQC, and HMBC spectra. The ^1^H-NMR spectral data for this compound was published early in 1976 [25], but recorded using a 100 MHz equipment. As in the ^1^H-NMR spectra of pipecolic derivative **8**, coupling constants for H-2 (*J*_2,3ax_ = 11.4, *J*_2,3eq_ = 4.2 Hz) for H-3_ax_ (*J*_3ax, 3eq_ = 14.9, *J*_3ax,4_ = 2.8 Hz) and H-3_eq_ (*J*_2,3eq_ = *J*_3eq,4_ ≈ 4.2) confirmed the S, R, and R configuration for C-2, C-4, and C-5, respectively, while NOE cross peaks between H-5–H-6', H-2 with H6', and H-3_ax_ with H-5 and H-4 suggested the chair conformation _4_C^N^. The optimized structure of compound **1** (Figure 5) was built into the Gaussian 9.0 platform and the energy minimized by the DFT method [28], which coincides with the NMR data obtained and configuration proposed. High resolution NMR (see Experimental section) and ESI-MS spectra reported in this work added more accurate information about this natural product structure, for which the NMR data were published a long time ago [25].

## 3. Experimental

### 3.1. General Experimental Procedures

Melting points were determined using a Stuart Scientific SMP3 melting point apparatus (Bibby Scientific Ltd, Staffordshire, UK). Analytical thin-layer chromatography (TLC) was performed on silica gel 60 F254 (Merck, Darmstadt, Germany) aluminum-supported plates (layer thickness 0.2 mm). Medium pressure column chromatography was performed with Silica gel (Kieselgel 60 H Merck, Darmstadt, Germany), 55 mm particle size, FMI QG 150 medium pressure lab pumps (Syosset, NY, USA) and Ace Glass Inc. medium pressure columns (Vineland, NJ, USA), using mixtures of n-hexane:ethyl acetate of different polarities as solvent system (flow rate: 5 mL/min.). Optical rotations were measured on an Autopol III automatic polarimeter (Rudolph Research Co., Hackettstown, NJ, USA). The NMR experiments (^1^H: 400.12 and 600.13 MHz; ^13^C: 100.25 and 150.09 MHz) were performed using either a Bruker Avance 400 or Bruker Avance II (Biospin, Rheinstetten, Germany) 600 UltraShield spectrometer with CD_3_OD or deuterated MeOD as solvent and TMS as internal standard. Optical rotations were measured on an Autopol III automatic polarimeter (Rudolph Research Co., Hackettstown, NJ, USA). IR spectra were measured using a Thermo Nicolet Nexus 470 FT-IR spectrometer (Thermo Nicolet, Madison, WI, USA) with KBr disks. The molecular weight was determined by low and high resolution with a mass spectrometer (Finnigan Mat 900 XLT, Thermo Fisher, Bremen, GmbH, Germany). Another mass spectrometer equipped with electrospray ion source and qToF analyzer MicrOTOF Q II (Bruker Daltonics Inc., Billerica, MA, USA) was used for HR-ESI-MS analysis. All reagents used for reactions were of analytical grade and purchased from Merck (Santiago, Chile) or Sigma-Aldrich (Santiago, Chile).

### 3.2. Synthesis of Methyl 3-deoxy-4,5:6,7-di-O-isopropylidene-2-O p-toluen-sulfonyl-d-gluco-heptonate (**4**)

To a stirred solution of compound **3** (1.38 g, 4.54 mmol) in anhydrous pyridine (2.7 mL) was added *p*-toluen-sulfonyl chloride (0.519 g, 4.54 mmol). After stirring at rt for 12 h, TLC (1:1, hexane/EtOAc) monitoring showed complete conversion of **3** (Rf = 0.58) into a less polar product (Rf = 0.75). The reaction mixture was concentrated and the residue purified by flash chromatography using as eluent mixtures of hexane/EtOAc (from 20:1 to 5:1), to afford crystalline **4** (1.99 g, 96%), mp 109 °C, [α]_D_: −42.10 (c 1.0, CH_3_OH). (+)HRESIMS: [M+H]^+^ = 459.1684, [M+Na]^+^ = 481.1489, (calcd for: C_21_H_31_O_9_S = 459.1683, and C_21_H_3O_NaO_9_S = 481.1502). ^1^H-NMR (CDCl_3_, 600 MHz) δ: 7.85 (d, 2H, *J* = 8.3 Hz, 2 H-Tosyl), 7.35 (d, 2 H, *J* = 8.3 Hz, 2 H-Tosyl), 5.10 (dd, 1 H, *J*_2,3_ = 10.8 *J*_2,3_ = 3.1 Hz, H-2) 4.10 (dd, 1 H, *J*_7,7'_ = 8.3 *J*_6,7_ = 5.9 Hz, H-7), 3.88 (dd, 1 H, *J*_7,7'_ = 8.3 *J*_6,7'_ = 5.1 Hz, H-7'), 3.92 (ddd, 1 H, *J*_5,6_ = 8.3, *J*_6,7'_ = 5.1, *J*_6,7_ = 5.9 Hz, H-6), 3.79 (ddd, 1 H, *J*_3,4_ = 2.2, *J*_3',4_= 10.4, *J*_4,5_ = 7.8 Hz, H-4), 3.44 (t, 1 H, *J*_4,5_ = 7.8, *J*_5,6_ = 8.3 Hz, H-5), 3.74 (s, 3 H, OCH_3_), 2.45 (s, 3 H, CH_3_-Tosyl), 2.28 (ddd, 1 H, *J*_2,3_ = 10.8, *J*_3,4_ = 2.2, *J*_3,3'_ = 14.2 Hz, H-3), 1.97 (ddd, 1 H, *J*_2,3'_ = 3.1, *J*_3',4_ = 10.4, *J*_3,3'_ = 14.2 Hz, H-3'), 1.36, 1.33, 1.32, 1.16 ((CH_3_)_2_C). ^13^C-NMR (150.09 MHz, CDCl_3_) δ: 169 (C-1), 145.0, 133.3, 129.7, 128.2 (C-aromatic), 109.7, 109.3 (Me_2_C), 81.1 (C-5), 76.7 (C-6), 75.2 (C-4), 75.2 (C-2), 67.7 (C-7), 52.7 (CH_3_O), 36.0 (C-3), 26.7, 26.6, 26.6, 25.1, ((CH_3_)_2_C), 21.7 (CH_3_-Tosyl).

### 3.3. Synthesis of Methyl 2-Azido-2,3-dideoxy-4,5:6,7-di-O-isopropylidene-d-manno-heptonate (**5**)

Compound **5** was obtained with 98% yield (0.68 g) from compound **4** (0.99 g, 2.16 mmol), as previously reported [27]. Physical properties and spectroscopic-spectrometric data for compound **5** were identical to those previously reported [27].

### 3.4. Synthesis of Methyl 2-Azido-2,3-dideoxy-4,5-O-isopropylidene-d-manno-heptonate (6)

A solution of **5** (0.68 g, 2.06 mmol) in acetic acid (25 mL) and water (0.3 mL) was stirred at 50 °C for 12 h. At the end of this time, TLC (1:2, hexane/EtOAc) monitoring showed complete conversion of **5** (Rf = 0.87) into a more polar product (Rf = 0.30). The mixture was concentrated under reduced pressure and the residue purified by medium pressure chromatography, using as eluent mixtures of hexane/EtOAc (from 5:1 to 1:7) to yield **6** (0.56 g, 94%).

### 3.5. Synthesis of (2S,4R,5R) 4,5-O-Isopropylidene-methyl-pipecolate (**8**)

A solution of **6** (0.56 g, 1.94 mmol) in MeOH (32 mL) was added to sodium periodate (0.48 g, 2.27 mmol) and stirred at room temperature (25 C) for 3 h. At the end of this time, TLC (1:1, hexane/EtOAc) monitoring showed complete conversion of **6** (Rf = 0.12) into a less polar product (**7**, Rf = 0.52). The reaction mixture was filtered, concentrated, and the syrupy residue dissolved in EtOAc (8 mL) with palladium black (10% Pd on carbon, 0.05 g); the sample was then hydrogenated at 45 psi until TLC (1:1, hexane/EtOAc) revealed total consumption of the starting material. After this time (12 h), the catalyst was removed by filtration with celite (2 g). The reaction mixture was concentrated and the residue purified by medium pressure chromatography using as eluent mixtures of hexane/EtOAc (from 5:1 to 1:7) to yield **8**, which crystallized from hexane-EtOAc 10:1 (0.11 g, 27%), mp 119 °C, [α]_D_ −47.62 (c 0.41, CHCl_3_), (+) HRESIMS [2M+H]^+^ = 431.2396. (calcd for: C_10_H_18_NO_4_ = 431.2472). ^1^H-NMR (CDCl_3_, 600 MHz) δ: 4.62 (ddd, 1 H, *J*_3',4_ = 8.6, J_4,5_ = 6.6, *J*_4,3_ = 4.2 Hz, H-4), 3. 87 (td, *J*_4,5_ = *J*_5,6_ ≈ 6.0, *J*_5,6'_ = 2.8 Hz, H-5), 3.75 (s, 3 H, OCH_3_) 3.31 (dd, 1H, *J*_2,3'_ = 4.4, *J*_2,3_ = 8.8 Hz, H-2), 3.07 (dd, 1H, *J*_5,6_ = 6.3, *J*_6,6'_ = 12.7 Hz, H-6), 2.51(dd, 1H, *J*_5,6'_ = 2.8, *J*_6',6_ = 12.7 Hz, H-6') 2.04 (ddd, 1 H, *J*_2,3'_ = 4.4, *J*_3',4_ = 8.8, *J*_3,3'_ = 14.0 Hz, H-3'), 2.03 (ddd, 1 H, *J*_2,3_ = 8.8, *J*_3,4_ = 4.0, *J*_3,3'_ = 14.0 Hz, H-3), 1.36, 1.33 (CH_3_)_2_C. ^13^C-NMR (150.09 MHz, D_2_O) δ: 175.4 (C-1), 107.9 ((CH_3_)_2_C), 79.85 (C-4), 74.48 (C-5), 58.2 (C-2), 35.89 (C-3), 49.1 (C-6), 27.5 ((CH_3_)_2_C), 27.3 ((CH_3_)_2_C).

### 3.6. Synthesis of (2S,4R,5R) 4,5 Dihydroxy-pipecolic Acid (**1**) from **8**

A solution of **8** (0.11 g, 0.51 mmol) was stirred into 2 mL trifluoroacetic acid-water (1:1), for 8 h at room temperature, when TLC analysis (1:1, hexane/EtOAc) showed consumption of all starting material and only one product with Rf = 0.4. Afterwards, the crude product was evaporated *in vacuo* and the residue dissolved in MeOH (1mL), and NaOH 0.1 M (1 mL) was added. TLC analysis (MeCN/EtOH/H_2_O/AcOH, 13:4:2:1, reactive to ninhidrine) showed only one blue spot with Rf = 0.2 after 5 h. The reaction mixture was concentrated, dissolved in water (1 mL) acidified with trifluoracetic acid (to pH = 6), and submitted to ion exchange chromatography with a Dowex 50W (H^+^) resin column (5 cm × 1 cm).The column was rinsed with water (5 mL) and eluted with pyridine 0.1 N (10 mL) to obtain oily **1** (0.06 g, 83%). [α]_D_ −5.4, (c 0.36, H_2_O), [α]_D_ −7.3, (c 0.18, CH_3_OH), (+) HRESIMS: [M+H]^+^ = 162.0763, [M+Na]^+^ = 184.0582, (calcd for: C_6_H_12_NO_4_ = 162.0766, and C_6_H_11_NNaO_4_ = 184.0580). ^1^H-NMR (600 MHz, D_2_O) δ: 3.92 (m, 1 H, *J*_3ax,4_ = *J*_4,5_ = 4.2 Hz, *J*_3eq,4_ = 2.8 Hz, H-4), 3.86 (dd, 1 H, *J*_2,3ax_ = 11.4, *J*_2,3eq_ = 4.2 Hz, H-2), 3.85 (m, 1 H. H-5), 3.32 (dd, 1H, *J*_5,6_ = 2.3, *J*_6,6'_ = 13.4 Hz, H-6), 3.19 (dd, 1 H, *J*_5,6'_ = 3.7, *J*_6,6'_ = 13.4 Hz, H-6'), 2.15 (ddd, 1 H, *J*_2,3ax_ = 11.4, *J*_3ax,4_ = 2.8, *J*_3ax,3eq_ = 14.9 Hz, H-3_ax_), 2.07 (dt, 1 H, *J*_2,3eq_ = *J*3_eq,4_ = 4.2, *J*_3ax,3eq_ = 14.9, H-3_eq_). ^13^C-NMR (150.09 MHz, D_2_O) δ: 173.35 (C-1), 65.4 (C-4), 64.9 (C-5), 53.5 (C-2), 44.2 (C-6) and 28.4 (C-3). Spectroscopic data was identical to that previously published [24].

### 3.7. Synthesis of Methyl 2-Benzyloxycarbonylamino-2,3-dideoxy-4,5:6,7-di-O-isopropylidene-d-manno-heptonate (**9**)

A solution of **5** (1 g, 3.03 mmol) in 20 mL EtOAc was hydrogenated at 30 psi with palladium black (0.1 g) until TLC (hexane/EtOAc 1:2) monitoring showed consumption of all the starting material (3 h) and a main spot with Rf = 0.25. The catalyst was removed by filtration on celite (4 g), the solution concentrated *in vacuo* to half of the volume (10 mL), and benzyl chloroformate (642 μL, 4.54 mmol) and triethyl amine (632 μL, 4.54 mmol) were added. After 5 h, the stirred reaction mixture showed a main spot in the TLC (Rf = 0.65) and the solution was concentrated *in vacuo*. The remaining residue was purified by flash chromatography with mixtures of hexane/EtOAc (from 20:1 to 5:1) to yield pure **9** (1.16 g, 88%). [α]_D_ +7.2 (c 0.33, CH_3_OH), (+) HRESIMS: [M+H]^+^ = 438.2122, [M+Na]^+^ = 460.1951, (calcd for: C_22_H_32_NO_8_ = 438.2122, and C_22_H_31_NNaO_8_ = 460.1942). ^1^H-NMR (CDCl_3_, 600 MHz) δ: 7.38 (br s, 5 H, Ph), 5.71 (d, 1H, *J*_2,NH_ = 6.5 Hz, NH), 5.15 (s, 2H, PhCH_2_O), 4.51 (q, 1 H, *J*_2,3_ = *J*_2,3'_ ≈ 6.3, *J*_2,NH_ = 6.5 Hz, H-2), 4.13 (dd, 1 H, *J*_7,7'_ = 8.5 *J*_6,7_ = 6.0 Hz, H-7), 3.93 (dd, 1 H, *J*_7,7'_ = 8.5 *J*_6,7'_ = 5.0 Hz, H-7'), 4.10 (m, 2 H, H-6, H-4, overlapped signals), 3.53 (t, 1 H, *J*_4,5_ = 8.9, *J*_5,6_ = 9.0 Hz, H-5), 3.76 (s, 3 H, OCH_3_), 2.38 (ddd, 1 H, *J*_2,3_ = 5.3, *J*_3,4_ = 2.8, *J*_3,3'_ = 14.5 Hz, H-3), 2.05 (ddd, 1 H, *J*_2,3'_ = 6.9, *J*_3',4_ = 9.8, *J*_3,3'_ = 14.5 Hz, H-3'), 1.41, 1.36, 1.34, 1.33 ((CH_3_)_2_C).^13 ^C-NMR (150.09 MHz, CDCl_3_) δ: 169.5 (C-1), 128.5, 128.1, 127.6, 127.4, 136.7 (C-aromatic), 110.5, 110.2 (Me_2_C), 81.3 (C-5), 77.2 (C-4), 76.9 (C-6), 67.7 (C-7), 66.9 (PhCH_2_O), 52.3 (CH_3_O), 52.2 (C-2), 35.5 (C-3), 27.0, 26.8, 26.6, 25.1 ((CH_3_)_2_C).

### 3.8. Synthesis of Methyl 2-Benzyloxycarbonylamino-2,3-dideoxy-4,5-O-isopropylidene-d-manno-heptonate (**10**)

A solution of **9** (1.16 g, 2.65 mmol) in acetic acid (45 mL) and water (0.6 mL) was stirred at 50 °C for 12 h. At the end of this time, TLC (1:2, hexane/EtOAc) monitoring showed complete conversion of **9** (Rf = 0.80) into a more polar product (Rf = 0.32). The mixture was concentrated under reduced pressure and the residue purified by flash chromatography, using as eluent mixtures of hexane/EtOAc (from 5:1 to 1:7) to yield **10** (0.86 g, 82%). [α]_D_ −6.5 (c 1.0, CH_3_OH), (+) HRESIMS: [M+H]^+^= 398.1810 (calcd for: C_19_H_28_NO_8_ = 398.1815. ^1^H-NMR (MeOD, 600 MHz) δ: 7.35 (m, 5 H, Ph), 5.10 (s, 2H, PhCH_2_O),4.42 (t, 1 H, *J*_2,3_ = 6.5, *J*_2,3'_ = 6.42 Hz, H-2), 4.12 (ddd, 1 H, *J*_3,4_ = 3.0, *J*_4,3'_ = 9.0, *J*_4,5_ = 7.5 Hz, H-4), 1 H, 3.71 (dd, 1 H, *J*_6,7_ = 3.4, *J*_7,7'_ = 11.10 Hz, H-7), 3.65 (t, 1 H, *J*_5,4_ = 7.5, *J*_5,6_ = 7.49 Hz, H-5), 3.60 (td, 1 H, *J*_6,5_ = 7.5, *J*_7,6_ = 3.4, *J*_7',6_ = 6.1 Hz, H-6), 3.55 (dd, 1 H, *J*_6,7'_ = 6.1, *J*_7,7'_ = 11.10 Hz, H-7'), 3.80 (s, 3 H, OCH_3_) 2.31 (ddd, 1 H, *J*_2,3_ = 8.79, *J*_3,4_ = 3.5, *J*_3,3'_ = 14.0 Hz, H-3), 2.03 (ddd, 1 H, *J*_2,3'_ = 8.0, *J*_3',4_ = 9.0, *J*_3,3'_ = 15.2 Hz, H-3'), 1.35, 1.32 ((CH_3_)_2_C). ^13^C-NMR (150.09 MHz, D_2_O) δ: 175.2 (C-1), 136.77, 128.07, 127.6, 127.4, (CH_2_C_6_H_5_), 108.9 (CH_3_)_2_C, 80.15 (C-5), 76.87 (C-4), 73.37 (C-6), 66.27 (CC_2_Ph), 63.57 (C-7), 52.02 (C-2), 35.95 (C-3), 26.08, 25.87 ((CH_3_)_2_C).

### 3.9. Synthesis of Methyl 2-Benzyloxycarbonyl-amino-2,3-dideoxy-4,5-O-isopropylidene-d-arabino-hexonate (**11**)

To a solution of **10** (0.86 g, 2.16 mmol) in MeOH (55 mL) was added sodium periodate (0.74 g, 3.46 mmol); this was stirred at room temperature (25 °C) for 4 h. When TLC (1:2, hexane/EtOAc) showed complete consumption of **10** and conversion into a less polar product (Rf = 0.35), the white solid formed was filtered, sodium cyanoborohydride (0.20 g, 3.18 mmol) was added, and a solution of HCl 0.1 N was added dropwise to maintain pH = 5. After 1 h of stirring at 0 °C, a main spot with Rf = 0.45 was detected by TLC (1:2, hexane/EtOAc). The solution was concentrated under reduced pressure, the salts filtere,d and the residue purified by flash chromatography using mixtures of hexane/EtOAc (from 10:1 to 1:2) as eluent to give **11** (0.78 g, 91%). [α]_D_ −10.16 (c 1.0, CHCl_3_), (+) HRESIMS: [M+H]^+^ = 368.1706, [M+Na]^+^ = 390.1524, (calcd for: C_18_H_26_NO_7_ = 368.1703, and C_18_H_25_NNaO_7_ = 390.1523). ^1^H-NMR (CDCl_3_, 600 MHz) δ: 7.37 (m, 5 H, H-aromatic), 5.73 (d, 1H, *J*_2,NH_ = 6.0 Hz, NH), 5.15 (s, 2H, PhCH_2_O), 4.49 (m, 2H, H-2, HO), 4.23 (dd, 1 H, *J*_3,4_ ≈ *J*_3',4_ ≈ *J*_4,5_ = 7.2 Hz, H-4), 4.08 (dd, 1H, *J*_5,6_ = 6.6, *J*_6,6'_ = 11.9 Hz, H-6), 3.95 (m, 2H, H-5, H-6'), 3.78 (s, 3 H, OCH_3_), 2.33 (ddd, 1 H, *J*_2,3_ = 8.82, *J*_3,4_ = 3.4, *J*_3,3'_ = 14.0 Hz, H-3), 2.03 (ddd, 1 H, *J*_2,3'_ = 8.2, *J*_3',4_ = 9.5, *J*_3,3'_ = 15.4 Hz, H-3'), 1.37, 1.34 ((CH_3_)_2_C).

^13^C-NMR (150.09 MHz, CDCl_3_) δ: 171.9 (C-1), 135.0-128.0 (C-aromatic), 110.7 (Me_2_C), 67.5 (PhCH_2_O), 80.9 (C-5), 75.6 (C-4), 74.2 (C-6), 52.7 (CH_3_O), 51.4 (C-2), 36.4 (C-3), 27.2, 26.7 ((CH_3_)_2_C).

### 3.10. Synthesis of Methyl 2-Benzyloxycarbonyl-amino-2,3-dideoxy-4,5-O-isopropylidene-6-O-p-toluen-sulfonyl-d-arabino-hexonate (**12**)

To a stirred solution of compound **11** (0.78 g, 2.13 mmol) in anhydrous pyridine (8.2 mL) was added *p*-toluen-sulfonyl chloride (0.56 g, 2.94 mmol). After stirring at rt for 12 h, TLC (1:1, hexane/EtOAc) monitoring showed complete conversion of **11** (Rf = 0.45) into a less polar product (Rf = 0.75); the mixture was then concentrated under reduced pressure and the residue purified by flash chromatography (hexane/EtOAc from 20:1 to 5:1), to afford oily **12** (0.97 g, 88%).

[α]_D_ +1.72 (c 1.0, CH_3_OH), (+) HRESIMS: [M+H]^+^ = 522.1798, [M+Na]^+^ = 544.1617, (calcd for: C_25_H_32_NO_9_S = 522.1792, and C_25_H_31_NNaO_9_S = 544.1611). ^1^H-NMR (CDCl_3_, 600 MHz) δ: 7.81 (d, 2 H, *J* = 8.3 Hz, 2 H-Tosyl), 7.39 (br s, 5 H, Ph), 7.37 (d, 2 H, *J* = 8.3 Hz, 2 H-Tosyl), 5.51 (d, 1H, *J*_2,NH_ = 6.5 Hz, NH), 5.15 (s, 2H, PhCH_2_O), 4.47 (q, 1 H, *J*_2,3_ = *J*_2,3'_ ≈ 6.0, *J*_2,NH_ = 6.5 Hz, H-2), 4.10 (d, 2H, *J*_5,6_ = 4.7, CH_2_-6), 3.95 (ddd, 1 H, *J*_3,4_ = 2.7, *J*_4,3'_ = 9.5, *J*_4,5_ = 7.0 Hz, H-4), 3.84 (dt, 1 H, *J*_4,5_ = 7.0, *J*_5,6_ = 4.7 Hz, H-5), 3.76 (s, 3 H, OCH_3_), 2,4 (s, 3 H, CH_3_-Tosyl), 2.19 (ddd, 1 H, *J*_2,3_ = 5.6, *J*_3,4_ = 2.7, *J*_3,3'_ = 14.5 Hz, H-3), 2.03 (ddd, 1 H, *J*_2,3'_ = 6.3, *J*_3',4_ = 9.5, *J*_3,3'_ = 14.5 Hz, H-3'), 1.37, 1.34 ((CH_3_)_2_C). ^13^C-NMR (150.09 MHz, CDCl_3_) δ: 169.7 (C-1), 130.0–128.0 (C-aromatic), 110.2 (Me_2_C), 78.0 (C-5), 74.6 (C-4), 68.5 (C-6), 67.1 (PhCH_2_O), 52.5 (CH_3_O), 52.0 (C-2), 35.1 (C-3), 27.0, 26.7 ((CH_3_)_2_C), 21.7 (CH_3_-Tosyl).

### 3.11. Synthesis of Compound 1 ((2S,4R,5R) 4,5 Dihydroxy-pipecolic acid) from **12**

A solution of **12** (0.97 g, 1.86 mmol) in EtOAc (17 mL) was added to 10% Pd on carbon (0.1 g) and hydrogenated at 45 psi. When TLC (1:1, hexane/EtOAc) revealed total consumption of the starting material (12 h), the catalyst was removed by filtration with celite (2 g). The crude product was evaporated *in vacuo *and to the residue we added trifluoracetic acid (3.7 mL) and water (3.7 mL), stirred overnight at rt for 12 h. After this time, the solution was evaporated to dryness, and water (10 mL) and K_2_CO_3_ (0.276 g, 2 mmol) were added. After 2 h of stirring, TLC (MeCN/EtOH/H_2_O/AcOH, 13:4:2:1) revealed total conversion of the previous compound (orange spot, Rf = 0.3) to a more polar one (blue spot reactive to ninhidrine, Rf = 0.12). The solution was evaporated to dryness, diluted with acidified water (to pH ≈ 5), submitted to ion exchange chromatography (Dowex 50W H^+^ resin column, 20 cm × 1 cm); then the column was rinsed with water (15 mL) and eluted with pyridine 0.1 N (20 mL) to obtain syrupy **1** (0.06 g, 88%), [α]_D_ −5.4, (c 0.36, H_2_O), [α]_D_ −7.3, (c 0.18, CH_3_OH), (+)HRESIMS: [M+H]^+^ = 162.0763 (calcd. for: C_6_H_12_NO_4_ = 162.0766). ^1^H-NMR (600 MHz, D_2_O) δ: 3.92 (m, 1 H, *J*_3ax,4_ = *J*_4,5_ = 4.2 Hz, *J*_3eq,4_ = 2.8 Hz, H-4) 3.86 (dd, 1 H, *J*_2,3ax_ = 11.4, *J*_2,3eq_ = 4.2 Hz, H-2), 3.85 (m, 1 H. H-5), 3.32 (dd, 1H, *J*_5,6_ = 2.3, *J*_6,6'_ = 13.4 Hz, H-6), 3.19 (dd, 1 H, *J*_5,6'_ = 3.7, *J*_6,6'_ = 13.4 Hz, H-6'), 2.15 (ddd, 1 H, *J*_2,3ax_ = 11.4, *J*_3ax,4_ = 2.8, *J*_3ax,3eq_ = 14.9 Hz, H-3_ax_), 2.07 (dt, 1 H, *J*_2,3eq_ = *J*3_eq,4_ = 4.2, *J*_3ax,3eq_ = 14.9, H-3_eq_). ^13^C-NMR (150.09 MHz, D_2_O) δ: 173.35 (C-1), 65.4 (C-4), 64.9 (C-5), 53.5 (C-2), 44.2 (C-6), and 28.4 (C-3). Spectroscopic data was identical to that previously published [24].

### 3.12. Computational Details

Calculations were carried out with the B3LYP hybrid functionals and 6-31+G(d) basis set. Full geometry optimizations and transition structure (TS) searches were carried out with the Gaussian 09 package 18 [28]. The possibility of different conformational isomers was taken into account for all structures. Frequency analyses were carried out at the same level used in the geometry optimizations, and the nature of the stationary points was determined in each case according to the appropriate number of negative eigenvalues of the Hessian matrix.

## 4. Conclusions

The amino acid (2*S*,4*R*,5*R*) 4,5 dihydroxy-pipecolic acid (**1**) was synthesized by two enantiospecific sequences, using d-gluco-heptono-1,4-lactone as a chiral template. The key step in the first approach involved the preparation of compound **8** through nucleophilic attack after catalytic hydrogenation of the azide group from aldehyde **7**, obtained by degradative oxidation of diol **6**. The second strategy enabled the construction of the piperidine ring by intramolecular substitution of the tosylate at C-6 by the amine group at C-2, produced by hydrogenation of carbamate **12**, after removal of the isopropylidene group with F_3_CCO_2_H. The latter and more efficient sequence involved 12 steps and gave a 19% overall yield, employing a commercially available and inexpensive sugar lactone.

## References

[1] Fadel A., Lahrache N. (2007). An efficient synthesis of enantiomerically pure (R)-pipecolic acid, (S)-proline, and their *N*-alkylated derivatives. J. Org. Chem..

[2] Hanson G.J., Vuletich J.L., Bedell L.J., Bono C.P., Howard S.C., Welphy J.K., Woulfe S.L., Zacheis M.L. (1996). Design of MHC Class II (DR4) ligands using conformationally restricted imino acids at p3 and p5. Bioorg. Med. Chem. Lett..

[3] Matsoukas J.M., Agelis G., Hendrelis J., Yamdagni R., Wu Q., Ganter R., Smith J.R., Moore D., Morre G.J. (1993). Synthesis and biological activities of angiotensin H, sarilesin and sarmesin analogues containing Aze or Pip at position 7. J. Med. Chem..

[4] Galeazzi R., Mobbili G., Orena M. (2004). Modelling and synthesis of conformationally resticted amino acids. Curr. Org. Chem..

[5] Ohara C., Takahashi R., Miyagawa T., Yoshimura Y., Kato A., Adachi I., Takahata H. (2008). Synthesis of all stereoisomers of 3-hydroxypipecolic acid and 3-hydroxy-4,5-dehydropipecolic acid and their evaluation as glycosidase inhibitors. Bioorg. Med. Chem. Lett..

[6] Fantur K., Hofer D., Schitter G., Steiner A.J., Pabst B.M., Wrodnigg T.M., Stütz A.E., Paschke E. (2010). DLHex-DGJ, a novel derivative of 1-deoxygalactonojirimycin with pharmacological chaperone activity in human GM1-gangliosidosis fibroblasts. Mol. Gen. Met..

[7] Boucheron C., Compain P., Martin O.R. (2006). A stereodivergent approach to 1-deoxynojirimycin, 1-deoxygalactonojirimycin and 1-deoxymannojirimycin derivatives. Tetrahedron Lett..

[8] Steiner A.J., Schitter G., Stütz A.E., Wrodnigg T.M., Tarling C.A., Withers S.G., Fantur K., Mahuran D., Paschke E., Tropak M. (2008). 1-Deoxygalactonojirimycin-lysine hybrids as potent d-galactosidase inhibitors. Bioorg. Med. Chem..

[9] Thorstensson F., Wångsell F., Kvarnström I., Vrang L., Hamelink E., Jansson K., Hallberg A., Rosenquist A., Samuelsson B. (2007). Synthesis of novel potent hepatitis C virus NS3 protease inhibitors: Discovery of 4-hydroxy-cyclopent-2-ene-1,2-dicarboxylic acid as a N-acyl-l-hydroxyproline bioisostere. Bioorg. Med. Chem..

[10] Stöckel-Maschek A., Stiebitz B., Koelsch R., Neubert K. (2005). Novel 3-amino-2-hydroxy acids containing protease inhibitors. Part 1: Synthesis and kinetic characterization as aminopeptidase P inhibitors. Bioorg. Med. Chem..

[11] Manning K.S., Lynn D.G., Shabanowitz J., Fellows L.E., Singh M., Schrire B.D. (1985). A glucuronidase inhibitor from the seeds of Baphia racemosa: Application of fast atom bombardment coupled with collision activated dissociation in natural product structure assignment. J. Chem. Soc. Chem. Commun..

[12] Mallick A., Kumari N., Roy R., Palanivel A., Vankar Y.D. (2014). A concise synthesis of (2R,3R)- and (2R,3S)-3-hydroxypipecolic acids, and total synthesis of (–)-deoxoprosopinine and (+)-2-epi-deoxoprosopinine from d-glycals. Eur. J. Org. Chem..

[13] Fleet G.W.J., Witty D.R. (1990). Synthesis of homochiral β-hydroxy-α-aminoacids [(2S,3R,4R)-3,4-dihydroxyproline and (2S,3R,4R)-3,4-dihydroxypipecolic aicd] and of 1,4-dideoxy-1,4-imino-d-arabinitol [DAB1] and fagomine [1,5-imino-1,2,5-trideoxy-d-arabino-hexitol]. Tetrahedron Asymmetry.

[14] Romeo J.T., Swain L.A., Bleecker A.B. (1983). Cis-4-hydroxypipecolic acid and 2,4-cis-4,5-trans-4,5-dihydroxypipecolic acid from Calliandra. Phytochemistry.

[15] Despontin J., Marlier M., Dardenn G. (1977). l-cis-5-hydroxypipecolic acid from seeds of Gymnocladus dioicus. Phytochemistry.

[16] Hatanaka S.-I., Kaneko S. (1977). Cis-5-hydroxy-L-pipecolic acid from Morus alba and Lathyrus japonicus. Phytochemistry.

[17] Kumar P.S., Baskaran S. (2009). A regioselective reductive cleavage of benzylidene acetal: Stereoselective synthesis of N-Boc-protected cis-(2R,3S)-3-hydroxy pipecolic acid. Tetrahedron Lett..

[18] Hoarau S., Fauchere J.L., Pappalardo L., Roumestant M.L., Viallefont P. (1996). Synthesis of enantiomerically pure (2R, 5S)- and (2R, 5R)-5-hydroxypipecolic acid from glycinate schiff bases. Tetrahedron Asymmetry.

[19] Botman P.N.M., Dommerholt F.J., de Gelder R., Broxterman Q.B., Schoemaker H.E., Rutjes F., Blaauw R.H. (2004). Diastereoselective synthesis of (2S,5R)-5-hydroxypipecolic acid and 6-substituted derivatives. Org. Lett..

[20] Le Corre L., Dhimane H. (2005). Synthesis of 5-substituted pipecolic acid derivatives as new conformationally constrained ornithine and arginine analogues. Tetrahedron Lett..

[21] Occhiato E.G., Scarpi D., Guarna A., Tabasso S., Deagostino A., Prandi C. (2009). A short and convenient synthesis of enantiopure cis- and trans-4-hydroxypipecolic acid. Synthesis.

[22] Di Nardo C., Varela O. (1999). Enantioselective synthesis of (2R,4S)-4-hydroxy-pipecolic acid from d-gluconoheptono-1,4-lactone. J. Org. Chem..

[23] Scarpi D., Bartali L., Casini A., Occhiato E.G. (2013). Complementary and stereodivergent approaches to the synthesis of 5-Hydroxy- and 4,5-dihydroxypipecolic acids from enantiopure hydroxylated lactams. Eur. J. Org. Chem..

[24] Shewry P.R., Fowden L. (1976). 4,5-Dihydroxypipecolic acids in the seed of Julbernardia isoberlinia and Brachystegia. Phytochemistry.

[25] Marlier M., Dardenne G., Casimir M. (1976). 2S-Carboxy-4R,5S-dihydroxypiperidine et 2S-carboxy-4S,5S-dihydroxypiperidine a partir de Derris elliptica. Phytochemistry.

[26] Uhrig M.L., Simirgiotis M.J., Varela O. (2010). Enantiospecific synthesis of the sugar amino acid (2S,5S)-5-(aminomethyl)-tetrahydrofuran-2-carboxylic acid. Tetrahedron Asymmetry.

[27] Gómez R.V., Kolender A.A., Varela O. (2006). Synthesis of polyhydroxy amino acids based on d- and l-alanine from d-glycero-d-gulo-heptono-1,4-lactone. Carbohydr. Res..

[28] Frisch M.J., Trucks G.W., Schlegel H.B., Scuseria G.E., Robb M.A., Cheeseman J.R., Scalmani G., Barone V., Mennucci B., Petersson G.A. (2009). Gaussian Software.

